# Potential mouth rinses and nasal sprays that reduce SARS-CoV-2 viral load: What we know so far?

**DOI:** 10.6061/clinics/2020/e2328

**Published:** 2020-11-10

**Authors:** Gabriel de Toledo Telles-Araujo, Raquel D’Aquino Garcia Caminha, Monira Samaan Kallás, Aytan Miranda Sipahi, Paulo Sérgio da Silva Santos

**Affiliations:** I Departamento de Cirurgia Estomatologia, Patologia e Radiologia, Escola de Odontologia de Bauru (FOB-USP), Universidade de Sao Paulo, Bauru, SP; II Unidade de Tratamento Intensivo, Hospital Sirio Libanes, Sao Paulo, SP, BR, Intensive Care Unit, Sírio Libanês Hospital, São Paulo, Brazil; III Clinica e Laboratorio de Gastroenterologia Experimental (LIM07), Hospital das Clinicas (HCFMUSP), Faculdade de Medicina, Universidade de Sao Paulo, Sao Paulo, SP, BR

Dear editor:

In parallel with the efforts of the global scientific community toward investigating the pathophysiology, prevention, and treatment of coronavirus disease (COVID-19), all medical specialties that deal with frontline care have readapted their care protocols to better treat patients and protect their teams when fighting against the pandemic.

Concerning COVID-19 transmission, publications have focused on the premise that saliva plays a central role in the transmission of severe acute respiratory syndrome coronavirus 2 (SARS-CoV-2) and that procedures performed in oral and nasopharyngeal areas can generate a large number of droplets and aerosols. However, in the absence of vaccines or effective therapies, it is crucial to explore existing treatments to reduce the SARS-CoV-2 viral load. Infection control measures are still the only option for reducing the number of new infections (1). These studies reinforce the importance of biosafety and cross-infection prevention protocols in limiting viral spread during these procedures (2-4).

On the basis of the few previously published studies that focused on understanding the potential effectiveness of antimicrobial solutions against COVID-19, in this study, we aimed to review publications on local control measures that contribute toward the reduction of SARS-CoV-2 viral load in patients with COVID-19, with the intent of making the host oral cavity and nasopharyngeal mucosa less contagious, controlling droplet transmission mainly to healthcare providers, and flattening the COVID-19 curve.

To assess the literature on the virucidal effect of antimicrobial solutions, a systematic review was carried out with an electronic search of the following databases: PubMed/Medline and Cochrane. To establish the search strategy, all studies had to address the following question: “What are the local measures to decrease the coronavirus viral load in the nasopharyngeal and oropharyngeal tracts?” A descripted search strategy was structured with Boolean operators (AND/OR/NOT) and the following keywords: (SARS-cov-2) OR (COVID-19) OR (coronavirus) AND (povidone-iodine) OR (chlorhexidine digluconate) OR (hydrogen peroxide) OR (oral rinse) OR (mouthwashes) OR (anti-infective agents) OR (PVP-I) OR (β-cyclodextrin) OR (Citrox) AND (saliva) OR (nasal cavity) OR (mouth) OR (oral cavity) OR (throat) OR (nasopharyngeal) OR (oropharyngeal). The search included published articles until August 10, 2020. In addition, the gray literature was also reviewed, including papers that eventually met the eligibility criteria upon discussion.

This systematic review was carried out in accordance with the Preferred Reporting Items for Systematic Reviews and Meta-Analyses guidelines (5). All studies met the criteria established by the Patient, Intervention, Comparison, and Outcome strategy, as follows: participants (P), patients with COVID-19; intervention (I), solutions with virucidal activity; control (C), patients not using antimicrobial solutions; and outcome (O), the reduction of salivary SARS-CoV-2 viral load.

The inclusion criteria were as follows: 1) *in vitro*, *in vivo*, and randomized clinical trials that addressed the use of mouthwashes or nasal sprays to reduce the viral load of SARS-CoV-2, 2) unlimited study period, and 3) having no language restriction. The exclusion criteria were as follows: 1) case reports and 2) systematic reviews.

Overall, 75 articles were identified in the selected databases: 65 studies in PubMed/Medline, eight in Cochrane, and two in the gray literature. The final sample included 11 papers that fulfilled all of the above-mentioned inclusion and exclusion criteria (Figure 1).

The data and outcomes obtained from these selected articles are listed in Table 1.

In healthcare settings, including hospital intensive care units (ICU) and dental offices, COVID-19 transmission because of the overabundance of SARS-CoV-2 in droplets of saliva released as aerosols is not traceable to an index patient because the particles remain airborne for some time and then settle over horizontal surfaces in rooms/offices (13-15). As there are no drugs or vaccines for COVID-19 available yet, local infection control measures are the only available alternatives to slow viral transmission/infection.

The Guideline for the Diagnosis and Treatment of Novel Coronavirus Pneumonia (the 5th edition) (16), released by the National Health Commission of the People’s Republic of China, concluded that chlorhexidine may not be effective in eliminating SARS-CoV-2. In addition, an *in vitro* study also revealed the inefficacy of chlorhexidine digluconate in killing human coronaviruses, such as those causing SARS and Middle East Respiratory Syndrome and the endemic human coronavirus (17).

However, a recently published study that evaluated SARS-CoV-2 dynamics in various body fluid specimens, such as saliva, oropharyngeal swabs, and nasopharyngeal swabs, concluded that viral load in the saliva can transiently be decreased for 2 h after using chlorhexidine mouthwash in COVID-19 patients (18). However, to better understand the effectiveness of chlorhexidine in decreasing the viral load, randomized controlled trials with a greater number of patients are still necessary.

On the basis of the outcomes of this review, we strongly recommend the use of povidone-iodine (PVP-I) as a pre-procedure mouth rinse and nasal spray to reduce the SARS-CoV-2 viral load in oral aerosols (19). In our opinion, PVP-I could be considered an adjunct to personal protective equipment during this pandemic. PVP-I is a simple, affordable, and practically innocuous intervention that has shown promising virucidal results in a few *in vitro* studies and in the first *in vivo* study. Its use at the lowest concentration (0.5%) and for the lowest contact time (15 s) led to the complete inactivation of SARS-CoV-2. Hence, it is indicated for patients and healthcare workers.

Although PVP-I showed better virucidal activity than that of hydrogen peroxide, we elucidated the fact that most of the studies were performed in an *in vitro* scenario, which does not take into account the impact of host immunity when using the solution (where the response to the agent would be different).

Although aerosols are not the major source of SARS-CoV-2 transmission, they are considered a potential risk of contamination among frontline workers. We are aware that it is not possible to eliminate all risks in a healthcare setting. However, as the viral load of the mucosa in the oral cavity, throat, and nose is high and anatomically integrated, recontamination will occur soon after rinsing. Thus, the literature recommends applying PVP-I every 2-3 hours, up to four times per day, in those who have suspected or confirmed SARS-CoV-2 infection and are undergoing high-risk procedures that involve aerosol production, such as orotracheal intubation, beyond the oral care administered in an ICU to patients under mechanical ventilation (20).

To date, the substances that have been suggested to potentially reduce the viral load in COVID-19 patients in the studies that we reviewed are primarily PVP-I, followed by hydrogen peroxide and chlorhexidine. We do not recommend the use of cyclodextrin combined with Citrox, as there is no evidence in the literature regarding its real impact on the SARS-CoV-2 viral load. Four randomized clinical trials are underway, which may help better formulate guidelines and strategies to minimize COVID-19 transmission.

## Figures and Tables

**Figure 1:**
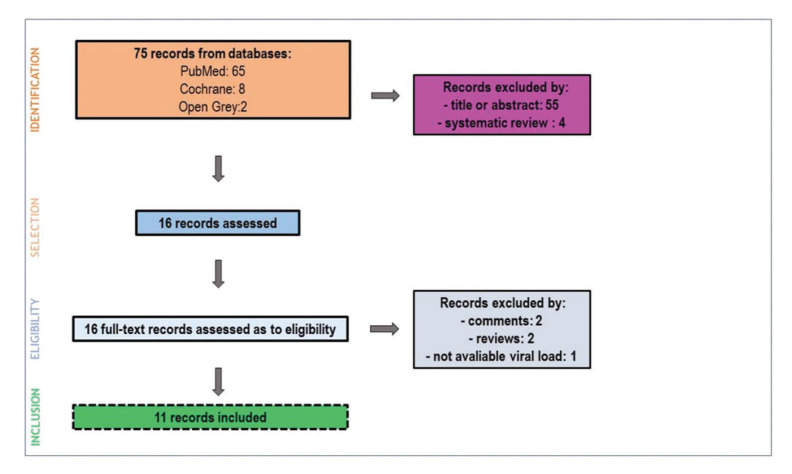
Flowchart of articles found in the PubMed/Medline, Cochrane, and the gray literature.

**Table 1:** Mouth rinses or nasal sprays to reduce SARS-CoV-2 viral load.

Authors, Year (Country)	Type of Publication Study	Sample (N)	Antimicrobial Solutions (Mouth rinse/Nasal spray)	Conclusions
Carrouel et al., 2020 [6] (France, Italy, Brazil, Spain)	Editorial (Multicenter)	NA	1. Citrox 2. Amphiphilic βCD 3. Cyclodextrins+Citrox	1. Citrox may reduce viral load 2. Amphiphilic βCD reduces viral load in oral cavity and nasal applications; hence, it may be considered in preventing viral transmission via the oropharyngeal route. 3. Clinical trials are still necessary to evaluate the benefits of βCD+Citrox in reducing the viral load of SARS-CoV-2.
Yoon et al., 2020 [7] (South Korea)	Clinical trial	2 patients	Chlorhexidine	Chlorhexidine mouthwash was effective in reducing the SARS-CoV-2 viral load in the saliva transiently (2 h).
Anderson et al., 2020 [8] (Singapore)	*In vitro* study	NA	PVP-I	Antiseptic solution (PVP-I 10%), skin cleanser (PVP-I 7.5%), gargle and mouth wash (PVP-I 1%), and throat spray (PVP-I 0.45%) achieved ≥ 99.99% virucidal activity against SARS-CoV-2 within 30 s.
Bidra et al., 2020 [9] (USA)	*In vitro* study	NA	PVP-I oral rinse (0.5%, 1.25%, and 1.5%) H_2_O_2_ aqueous solutions (3% and 1.5%)	PVP-I oral antiseptic rinse at all three concentrations completely inactivated SARS-CoV-2. H_2_O_2_ solutions at concentrations of 1.5% and 3.0% showed minimal virucidal activity after 15 s and 30 s of contact time.
Bidra et al., 2020 [10] (USA)	*In vitro* study	NA	PVP-I oral rinse (0.5%, 1.25%, and 1.5%) Positive control - Ethanol (70%) Negative control - Water	PVP-I oral antiseptics, at all tested concentrations, completely inactivated SARS-CoV-2 within 15 s of contact. Ethanol 70% was only able to inactivate the virus at 30 s of contact.
Lamas et al., 2020 [11] (Spain)	*In vivo* study	4 patients	PVP-I (1%)	In two of the four patients, PVP-I resulted in a significant drop in viral load, which remained for at least 3 h.
Liang et al., 2020 [12] (China, USA)	*In vitro* study	NA	PVP-I eye drop (gel forming) PVP-I nasal spray (gel forming)	Dose- and time-dependent inactivation of SARS-CoV-2 was observed in both the cases.
NCT04410159	Clinical Trial	NR	Povidone-iodine *versus* essential oil *versus* tap-water gargling for COVID-19 patients	NR
NCT04409873	Clinical Trial	NR	Antiseptic mouthwash/pre-procedural rinse on SARS-CoV-2 load (COVID-19)	NR
NCT04449965	Clinical Trial	60	Betadine sinonasal rinses, Betadine mouth gargle, and 6% PVP‐I gel forming nasal spray	NR
NCT04347954	Clinical Trial	NR	PVP-I nasal sprays and SARS-CoV-2 nasopharyngeal titers (for COVID-19)	NR

LEGEND: NA=not applicable; Citrox=combination of natural bioflavonoids extracted from citrus fruits; βCD=β-cyclodextrins; NR=not reported; PVP-I=povidone-iodine; H_2_O_2_=hydrogen peroxide.

## References

[1] Volgenant CMC, Persoon IF, Ruijter RAG, Soet JJ (2020). Infection control in dental health care during and after the SARS‐CoV‐2 outbreak. Oral Dis.

[2] Sabino-Silva R, Jardim ACG, Siqueira WL (2020). Coronavirus COVID-19 impacts to dentistry and potential salivary diagnosis. Clin Oral Investig.

[3] Peng X, Xu X, Li Y, Cheng L, Zhou X, Ren B (2020). Transmission routes of 2019-nCoV and controls in dental practice. Int J Oral Sci.

[4] Xu H, Zhong L, Deng J, Peng J, Dan H, Zeng X (2020). igh expression of ACE2 receptor of 2019-nCoV on the epithelial cells of oral mucosa. Int J Oral Sci.

[5] Moher D, Liberati A, Tetzlaff J, Altman DG, PRISMA Group (2009). Preferred reporting items for systematic reviews and meta-analyses: the PRISMA statement. PLoS Med.

[6] Carrouel F, Conte MP, Fisher J, Gonçalves LS, Dussart C, Llodra JC (2020). COVID-19: A Recommendation to Examine the Effect of Mouthrinses with β-Cyclodextrin Combined with Citrox in Preventing Infection and Progression. J Clin Med.

[7] Yoon JG, Yoon J, Song JY, Yoon SY, Lim CS, Seong H (2020). Clinical Significance of a High SARS-CoV-2 Viral Load in the Saliva. J Korean Med Sci.

[8] Anderson DE, Sivalingam V, Kang AEZ, Ananthanarayanan A, Arumugam H, Jenkins TM (2020). Povidone-Iodine Demonstrates Rapid *In Vitro* Virucidal Activity Against SARS-CoV-2, The Virus Causing COVID-19 Disease. Infect Dis Ther.

[9] Bidra AS, Pelletier JS, Westover JB, Frank S, Brown SM, Tessema B (2020). Comparison of *In Vitro* Inactivation of SARS CoV-2 with Hydrogen Peroxide and Povidone-Iodine Oral Antiseptic Rinses. J Prosthodont.

[10] Bidra AS, Pelletier JS, Westover JB, Frank S, Brown SM, Tessema B (2020). Rapid *In-Vitro* Inactivation of Severe Acute Respiratory Syndrome Coronavirus 2 (SARS-CoV-2) Using Povidone-Iodine Oral Antiseptic Rinse. J Prosthodont.

[11] Martínez Lamas L, Diz Dios P, Pérez Rodríguez MT, Del Campo Pérez V, Cabrera Alvargonzalez JJ, López Domínguez AM (2020). Is povidone iodine mouthwash effective against SARS-CoV-2? First *in vivo* tests. Oral Dis.

[12] Liang B, Yuan X, Wei G, Wang W, Zhang M, Peng H (2020). *In-Vivo* Toxicity Studies and *In-Vitro* Inactivation of SARS-CoV-2 by Povidone-iodine *In-situ* Gel Forming Formulations. bioRxiv.

[13] Ferretti L, Wymant C, Kendall M, Zhao L, Nurtay A, Abeler-Dörner L (2020). Quantifying SARS-CoV-2 transmission suggests epidemic control with digital contact tracing. Science.

[14] Jones RM, Brosseau LM (2015). Aerosol transmission of infectious disease. J Occup Environ Med.

[15] Liu L, Wei Q, Alvarez X, Wang H, Du Y, Zhu H (2011). Epithelial cells lining salivary gland ducts are early target cells of severe acute respiratory syndrome coronavirus infection in the upper respiratory tracts of rhesus macaques. J Virol.

[16] Office of the National Health and Health Commission Office of the State Administration of Traditional Chinese Medicine Guideline for the Diagnosis and Treatment of Novel Coronavirus Pneumonia - the 5th edition [Internet].

[17] Kampf G, Todt D, Pfaender S, Steinmann E (2020). Persistence of coronaviruses on inanimate surfaces and their inactivation with biocidal agents. J Hosp Infect.

[18] Yoon JG, Yoon J, Song JY, Yoon SY, Lim CS, Seong H (2020). Clinical Significance of a High SARS-CoV-2 Viral Load in the Saliva. J Korean Med Sci.

[19] Marui VC, Souto MLS, Rovai ES, Romito GA, Chambrone L, Pannuti CM (2019). Efficacy of preprocedural mouthrinses in the reduction of microorganisms in aerosol: A systematic review. J Am Dent Assoc.

[20] Mady LJ, Kubik MW, Baddour K, Snyderman CH, Rowan NR (2020). Consideration of povidone-iodine as a public health intervention for COVID-19: Utilization as &quot;Personal Protective Equipment&quot; for frontline providers exposed in high-risk head and neck and skull base oncology care. Oral Oncol.

